# Adjuvant treatment with interleukin-2- and interferon-alpha2a-based chemoimmunotherapy in renal cell carcinoma post tumour nephrectomy: Results of a prospectively randomised Trial of the German Cooperative Renal Carcinoma Chemoimmunotherapy Group (DGCIN)

**DOI:** 10.1038/sj.bjc.6602443

**Published:** 2005-03-08

**Authors:** J Atzpodien, E Schmitt, U Gertenbach, P Fornara, H Heynemann, A Maskow, M Ecke, H H Wöltjen, H Jentsch, W Wieland, T Wandert, M Reitz, DGCIN – German Cooperative Renal Carcinoma Chemo-Immunotherapy Trials Group

**Affiliations:** 1 Fachklinik Hornheide an der Universität Münster, Internistische Onkologie, Dorbaumstr. 300, 48157 Münster, Germany; 2 Europäisches Institut für Tumor Immunologie und Prävention, Gotenstr. 152, 53175 Bonn, Germany; 3 Kreiskrankenhaus Aschersleben, Urologische Klinik, Eislebender Str. 7a, 06449 Aschersleben, Germany; 4 Allgemeines Krankenhaus der Stadt Hagen, Urologische Klinik, Grünstr. 35, 58095 Hagen, Germany; 5 Klinikum der Martin-Luther-Universität, Urologische Klinik, Ernst-Grube-Str. 40, 06120 Halle a.d. Saale, Germany; 6 Universitätsklinikum Leipzig, Klinik und Poliklinik für Urologie, Liebigstr. 21, 04103 Leipzig, Germany; 7 Städtisches Klinikum Magdeburg, Urologie, Birkenallee 34, 39002 Magdeburg, Germany; 8 Klinikum Minden, Hämatologie/Onkologie, Portastr. 7-9, 32423 Minden, Germany; 9 Klinikum Ernst-von Bergmann, Klinik für Urologie, Charlottenstr. 72, 14467 Potsdam, Germany; 10 Caritas Krankenhaus St Josef, Urologie, Landshuterstr. 65, 93053 Regensburg, Germany

**Keywords:** adjuvant, immunotherapy, renal cell carcinoma

## Abstract

We conducted a prospectively randomised clinical trial to investigate the role of adjuvant outpatient immunochemotherapy administered postoperatively in high-risk patients with renal cell carcinoma. In total, 203 renal carcinoma patients' status post radical tumour nephrectomy were stratified into three risk groups: patients with tumour extending into renal vein/vena cava or invading beyond Gerota's fascia (pT3b/c pN0 or pT4pN0), patients with locoregional lymph node infiltration (pN+), and patients after complete resection of tumour relapse or solitary metastasis (R0). Patients were randomised to undergo either (A) 8 weeks of outpatient subcutaneous interleukin-2 (sc-rIL-2), subcutaneous interferon-alpha2a (sc-rIFN-*α*2a), and intravenous 5-fluorouracil (iv-5-FU) according to the standard Atzpodien regimen (Atzpodien *et al*, 2004) or (B) observation. Two-, 5-, and 8-year survival rates were 81, 58, and 58% in the treatment arm, and 91, 76, and 66% in the observation arm (log rank *P*=0.0278), with a median follow-up of 4.3 years. Two, 5-, and 8-year relapse-free survival rates were calculated at 54, 42, and 39% in the treatment arm, and at 62, 49, and 49% in the observation arm (log rank *P*=0.2398). Stage-adapted subanalyses revealed no survival advantages of treatment over observation, as well. Our results established that there was no relapse-free survival benefit and the overall survival was inferior with an adjuvant 8-week-outpatient sc-rIL-2/sc-rIFN-*α*2a/iv-5-FU-based immunochemotherapy compared to observation in high-risk renal cell carcinoma patients following radical tumour nephrectomy.

During the last decades, renal cell cancer has been increasing in incidence in North America and Europe, with approximately one-third having metastatic disease at the time of diagnosis. Patients with locally advanced renal cell carcinoma are at high risk of recurrence, since relapse rates range from 50 to 85% depending on tumour (T) stage and nodal (N) status, and reach close to 100% in recurrent disease patients who have undergone R0 resection (Fleischmann *et al*, 1997; Messing *et al*, 2003).

Until now, no adjuvant treatment including radiation, chemotherapy, or immunotherapy of locally advanced renal cell carcinoma has shown satisfactory results (Clark *et al*, 2003).

Promising results in the therapy of stage IV metastatic renal cell carcinoma were reported using interleukin-2 (rIL-2) given intravenously or subcutaneously as outpatient therapy alone or in combination with interferon-*α* (rINF-*α*) yielding objective response rates between 19 and 31% (Sleijfer *et al*, 1992; Rosenberg *et al*, 1994; Atzpodien *et al*, 1995). Hereby, cytokine outpatient therapy regimens (Atzpodien *et al*, 1995) showed highly reduced systemic toxicities as compared to i.v. bolus infusions. The combination of subcutaneous cytokines with the chemotherapeutic i.v. 5-fluorouracil (5-FU) further enhanced antineoplastic activity achieving objective response rates between 18 and 39% (Lopez-Hänninen *et al*, 1996; Dutcher *et al*, 2000; Atzpodien *et al*, 2001, 2004). Based on its efficacy in metastatic renal cell carcinoma, we hypothesized that outpatient sc-rIL-2/sc-rIFN-*α*2a/iv-5-FU-based immunochemotherapy according to the standard Atzpodien regimen (Atzpodien *et al*, 2004) might extend progression-free and/or overall survival of high-risk renal cell carcinoma patients in the postsurgical adjuvant setting.

We, therefore, performed a prospective randomized clinical study to compare the efficacy of sc-rIL-2/sc-rIFN-*α*2a/iv-5-FU *vs* observation as an adjuvant approach in high-risk renal carcinoma patients.

## PATIENTS AND METHODS

### Patients

Between October 1993 and February 2002, 203 high-risk patients with resected renal cell carcinoma were stratified into three risk groups: (1) patients with tumour extending into renal vein/vena cava or invading beyond Gerota's fascia (pT3b/c pN0 or pT4pN0; *n*=77), (2) patients with locoregional lymph node infiltration (pN+, *n*=36), and (3) patients after complete resection of tumour relapse or solitary metastasis (pR0; *n*=90); spontaneous 5-year systemic survival rates were 84, 70, and 71% in pT3b/c pN0 or pT4pN0 patients, pN+ patients, and R0 patients, respectively. Systemic pretreatment included, chemotherapy (*n*=1), immunotherapy (*n*=10), and naturopathic therapy (*n*=1) (Table 1).

Criteria for entry into the study were: histologically confirmed renal cell carcinoma (pT3b/c pN0 or pT4pN0; pN+; R0), age between 18 and 80 years; white blood cell count ⩾3500 *μ*l^−1^; platelet count ⩾100 .000 *μ*l^−1^; hematocrit ⩾30%; serum bilirubin ⩽1.25, and creatinine ⩽1.5 of the upper normal limit; Karnofsky performance status ⩾80%; no evidence of congestive heart failure, no severe coronary artery disease, no cardiac arrhythmias, no clinically symptomatic CNS disease or seizure disorders, no human immunodeficiency virus infection, no evidence of chronic active hepatitis, no concomitant corticosteroid therapy. In all patients treated, no chemotherapy or immunmodulatory treatment had been performed during the previous 4 weeks. Also, pregnant and lactating women were excluded.

This study was approved by the institutional review board of the Medizinische Hochschule Hannover. Upon written receipt of patient prestudy evaluation, randomisation was performed; 135 patients were assigned to arm A, and 68 patients were assigend to arm B.

### Regimens

Patients randomised to adjuvant therapy (arm A) received one 8-week treatment cycle of sc rIFN-*α*2a (Roferon®, Hoffmann-La Roche; Grenzach-Wyhlen, Germany) (5 × 10^6^ IU m^−2^, day 1, weeks 1+4; days 1, 3, 5, weeks 2+3; 10 × 10^6^ IU m^−2^, days 1, 3, 5, weeks 5–8), sc rIL-2 (Proleukin®, Chiron, Emeryville, CA, USA) (10 × 10^6^ IU m^−2^, twice daily days 3–5, weeks 1+4; 5 × 10^6^ IU m^−2^, days 1, 3, 5, weeks 2 + 3) and iv 5-FU (1000 mg m^−2^, day 1, weeks 5–8). Patients at an age of 60 years and older received a 20% dose reduction of sc IL-2 to avoid toxic complications. Concomitant medication was given as needed to control adverse effects of immunochemotherapy. Patients randomised to undergo observation (arm B/control) received no adjuvant therapy. In the event of relapse, all patients were offered individual care outside the present study.

### Assessment of survival

Survival was measured from start of therapy to date of death or to the last known date to be alive. In case of progression upon first re-evaluation after 8 weeks, relapse-free survival was calculated at 0 months. All patients had to be followed up for survival for at least 2 years as cutoff.

### Statistical analysis

The statistical end points in our analysis were (1) relapse-free survival (primary end point) and, (2) overall survival of patients. The probability of relapse-free survival and overall survival was plotted over time according to the method of Kaplan and Meier (1958).

The potential 2-year –relapse-free survival rates were hypothesised to show a 20% advantage of Arm A over Arm B (90 *vs* 70%). Using an *α* of 0.05 (one-sided), a sample size of 59 patients plus 20% potential drop outs per arm was needed to have 80% power to statistically establish the assumed difference in relapse-free survivals. The present 2 : 1 (treatment *vs* observation) randomisation was capable of meeting these statistical end points.

Statistical significance was assessed using the log rank test. For statistical analysis, the SPSS software for Windows (SPSS, Inc., Chicago, IL, USA) was applied.

## RESULTS

In total, 203 stratified renal carcinoma patients were prospectively randomised to undergo either an 8-week treatment with sc rIL-2, sc rIFN-*α*2a, and iv 5-FU (arm A, *n*=135 patients) or to undergo observation (arm B, *n*=68 patients).

### Overall survival

**Figure 1:**
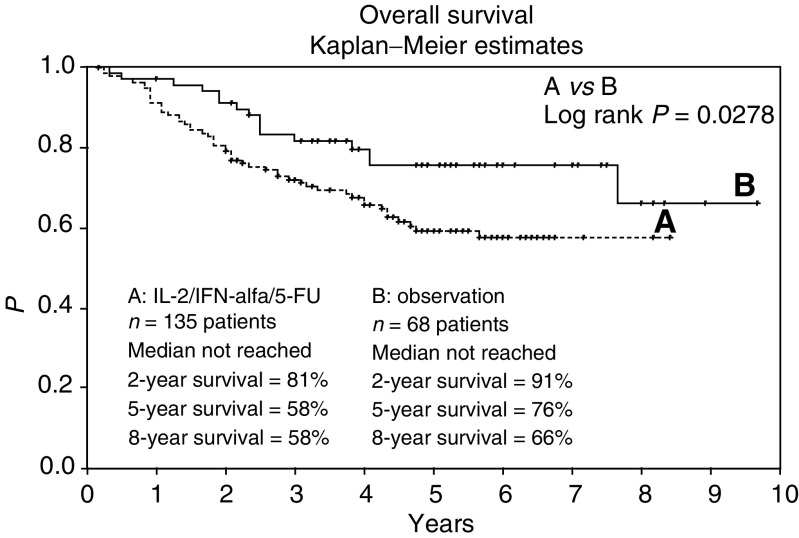
Overall survival for all 203 patients receiving (**A**) sc interleukin-2, sc interferon-*α*, and iv 5-fluorouracil, or (**B**) observation. Plots were generated by the Kaplan–Meier method.

After a median follow-up of 4.3 years (range, 0.2–9.7 years), 82 patients (61%) in arm A (sc-rIL-2/sc-rIFN-*α*2a/iv-5-FU) and 51 patients (75%) in arm B (observation) continued to be alive. No therapy-induced toxic deaths occurred. Two-, 5-, and 8-year survival probabilities were 81, 58, and 58% on the treatment arm, and 91, 76, and 66% on the observation arm (Figure 1). Overall survival was significantly decreased (log rank *P*=0.0278) after treatment with immunochemotherapy (range, 0.2–8.4 years), when compared with the control (range, 0.3–9.7 years) (Figure 1).

Stage-related (pT3b/c pN0 or pT4pN0, pN+, and R0) analysis revealed no survival advantages of treatment over observation in patient subgroups.

### Relapse-free survival

**Figure 2:**
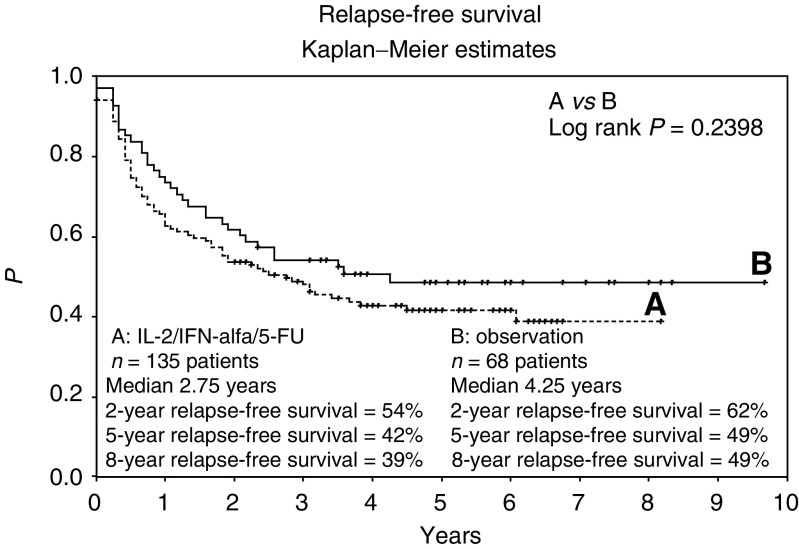
Relapse-free survival for all 203 patients receiving (**A**) sc interleukin-2, sc interferon-*α*, and iv 5-fluorouracil, or (**B**) observation. Plots were generated by the Kaplan–Meier method.

A total of 77 patients (57%) in arm A (sc-rIL-2/sc-rIFN-*α*2a/iv-5-FU) and 34 patients (50%) in arm B (observation) exhibited tumour progression at last follow-up. Two, 5-, and 8-year relapse-free survival probabilities were calculated at 54, 42, and 39% on the treatment arm, with a median relapse-free survival of 2.75 years (range, 0–8.2 years), and at 62, 49, and 49% on the observation arm, with a median relapse-free survival of 4.25 years (range, 0–9.7 years) (log rank *P*=0.2398)(Figure 2). Thus, the statistical hypotheses could not be established.

Within the three stratification groups (pT3b/c pN0 or pT4pN0, pN+, and R0), median relapse-free survival did not differ significantly between arm A (sc-rIL-2/sc-rIFN-*α*2a/iv-5-FU) and arm B (observation).

## DISCUSSION

In this prospectively randomised trial, we reported the results of 203 tumour-free renal cell carcinoma patients after surgery of locally advanced or distant metastatic disease who underwent an adjuvant treatment with sc-rIL-2/sc-rIFN-*α*2a/iv-5-FU (arm A) or underwent observation (arm B).

We demonstrated that high-risk renal cell carcinoma patients did not benefit from an eight-week postoperative adjuvant immunochemotherapy, both with respect to relapse-free survival and with respect to overall survival. Thus, the primary relapse-free survival end point of the current trial was not reached.

The lack of sc-rIL-2/sc-rIFN-*α*2a/iv-5-FU-related survival benefit in the adjuvant renal carcinoma therapy was disappointing, particularly when compared to the established efficacy in metastatic renal cell carcinoma patients (Lopez-Hänninen *et al*, 1996; Elias *et al*, 1999; Atzpodien *et al*, 2001, 2004).

However, recent studies employing *α*-IFN-based therapy in the adjuvant setting for renal cell carcinoma patients also did not result in significant improvement regarding relapse-free survival and overall survival (Pizzocaro *et al*, 2001; Messing *et al*, 2003).

In studies of Pizzocaro *et al* (2001), an adjuvant treatment with IFN-alfa2b demonstrated a decrease in relapse rates in 26 patients with extensive nodal disease (pN2/pN3), but an increase in recurrence rates in node negative renal carcinoma patients.

Using tumour vaccination protocols with no interleukin-2 or *α*-interferon, Repmann *et al* (2003) and Jocham *et al* (2004) were the first investigators reporting positive adjuvant treatment results in locally advanced renal cell carcinoma.

Overall, the present prospectively randomised clinical trial reported here established that adjuvant 8-week outpatient sc rIL-2/sc rIFN-*α*2a/iv 5-FU-based immunochemotherapy was inferior to observation when administrated to high-risk patients with resected renal cell carcinoma. It should be noted, though, that patients did not benefit from therapy, but indeed may have been harmed. This could be with toxicity, however, in the current trial toxicity was not systematically studied; this should also be subject of future trials.

## Figures and Tables

**Table 1:** Patients characteristics and pretreatment

	**Treatment**		
**Characteristics**	**Arm A^a^ (*n*)**	**Arm B^a^ (*n*)**	**All patients (*n*)**
*Entered*	135	68	203
			
*Age (years)*			
Median	59	60	59
Range	31–77	38–77	31–77
			
*Sex*			
Male	97	54	151
Female	38	14	52
			
*Radical nephrectomy*	135	68	203
			
*Stratum*			
pT3b/c pN0 or T4pN0	50	27	77
pN+	28	8	36
R0	57	33	90
Local	9	4	13
Lymph nodes	5	5	10
Organ metastases	37	22	59
Site unknown	6	2	8
			
*Histological subtype*			
Clear cell	84	41	125
Granular	3	2	5
Sarkomatoid	2	1	3
Papillary	1	1	2
Mixed	22	8	30
Unknown	23	15	38
			
*Systemic pretreatment*			
Chemotherapy	0	1	1
Immunotherapy^b^	4	6	10
Naturopathic therapy	1	0	1
Unknown	6	0	6

a Arm A (8 weeks of sc-rIL-2/sc-rIFN-*α* /iv 5-FU); Arm B (observation).

b Including IL-2/IFN-*α*2, IL-2/Vaccine, and Vaccine.

sc-rIL-2=subcutaneous interleukin-2; sc-rIFN-*α*2=subcutaneous interferon-alpha2a, and iv-5-FU=intravenous 5-fluorouracil.

## References

[bib1] Atzpodien J, Kirchner H, Duensing S, Lopez Hanninen E, Franzke A, Buer J, Probst M, Anton P, Poliwoda H (1995) Biochemotherapy of advanced metastatic renal-cell carcinoma: results of the combination of interleukin-2, alpha-interferon, 5-fluorouracil, vinblastine, and 13-*cis*-retinoic acid. World J Urol 13: 174–177 7550391 10.1007/BF00184875

[bib2] Atzpodien J, Kirchner H, Illiger HJ, Metzner B, Ukena D, Schott H, Funke PJ, Gramatzki M, von Jürgenson S, Wandert T, Patzelt T, Reitz M (2001) IL-2 in combination with IFN-*α* and 5-FU *vs* tamoxifen in metastatic renal cell carcinoma: long-term results of a controlled randomized clinical trial. Br J Cancer 85(8): 1130–1136 11710825 10.1054/bjoc.2001.2076 PMC2375150

[bib3] Atzpodien J, Kirchner H, Jonas U, Bergmann L, Schott H, Heynemann H, Fornara P, Loening SA, Roigas J, Müller SC, Bodenstein H, Pomer S, Metzner B, Rebmann U, Oberneder R, Siebels M, Wandert T, Puchberger T, Reitz M (2004) Interleukin-2- and interferon-alpha2a-based immuno-chemotherapy in advanced renal cell carcinoma: results of a prospectively randomized Trial of The German Cooperative Renal Carcinoma Chemoimmunotherapy Group (DGCIN). J Clin Oncol 22(7): 1188–1194 14981107 10.1200/JCO.2004.06.155

[bib4] Clark JI, Atkins MB, Urba WJ, Creech S, Figlin RA, Dutcher JP, Flaherty L, Sosman JA, Logan TF, White R, Weiss GR, Redman BG, Tretter CPG, McDermott D, Smith JW, Gordon MS, Margolin KA (2003) Adjuvant high-dose bolus interleukin-2 for patients with high-risk renal cell carcinoma: a Cytokine Working Group Randomized Trial. J Clin Oncol 21(16): 3133–3140 12810695 10.1200/JCO.2003.02.014

[bib5] Dutcher JP, Logan T, Gordon M, Sosman J, Weis G, Margolin K, Plasse T, Mier J, Lotze M, Clark J, Atkins M (2000) Pase II trial of interleukin-2, interferon-alfa, and 5-fluorouracil in metastatic renal cell cancer: a Cytokine Working Group Study. Clin Cancer Res 6: 3442–3450 10999727

[bib6] Elias L, Binder M, Mangalik, Clark D, Morrison B, Altobelli KK, Smith A (1999) Pilot trial of infusional 5-fluorouracil, interleukin-2, and subcutaneous interferon-alpha for advanced renal cell carcinoma. Am J Clin Oncol 22(2): 156–161 10199450 10.1097/00000421-199904000-00010

[bib7] Fleischmann J, Alyskewycz M, Flanigan RC (1997) Stage III renal cell carcinoma. Staging subcategories and prognosis: presented at American Urology Association Annual Meeting; 10–12 April; New Orleans, LA

[bib8] Jocham D, Richter A, Hoffmann L, Iwig K, Fahlenkamp D, Zartewski G, Schmitt E, Dannenberg T, Lehmacher W, von Wietersheim J, Doehn C (2004) Adjuvant autologous renal tumour cell vaccine and risk of tumour progression in patients with renal-cell carcinoma after radical nephrectomy: phase III, randomised controlled trial. Lancet 363(9409): 594–599 14987883 10.1016/S0140-6736(04)15590-6

[bib9] Kaplan EL, Meier P (1958) Nonparametric estimation from incomplete observations. J Am Stat Ass 53: 457

[bib10] Lopez-Hänninen EH, Kirchner H, Atzpodien J (1996) Interleukin-2 based home therapy of metastatic renal cell carcinoma: risks and benefits in 215 consecutive single institution patients. J Urol 155: 19–25 7490829

[bib11] Messing EM, Manola J, Wilding G, Propert K, Fleischmann J, Crawford ED, Pontes JE, Hahn R, Trump D (2003) Phase III study of intereferon alfa-NL as adjuvant treatment for resectable renal cell carcinoma: an Eastern Cooperative Oncology Group/Intergroup Trial. J Clin Oncol 21(7): 1214–1222 12663707 10.1200/JCO.2003.02.005

[bib12] Pizzocaro G, Piva L, Colavita M, Ferri S, Artusi R, Boracchi P, Parmiani G, Marubini E (2001) Interferon adjuvant to radical nephrectomy in Robson stages II and III renal cell carcinoma: a multicentric randomized study. J Clin Oncol 19(2): 425–431 11208835 10.1200/JCO.2001.19.2.425

[bib13] Repmann R, Goldschmidt AJ, Richter A (2003) Adjuvant therapy of renal cell carcinoma patients with an autologous tumor cell lysate vaccine: a 5-year follow-up analysis. Anticancer Res 23(2A): 969–974 12820332

[bib14] Rosenberg SA, Yang JC, Topalian SL, Schwartzentruber DJ, Weber JS, Parkinson DR, Seip CA, Einhorn JH, White DE (1994) Treatment of 283 consecutive patients with metastatic melanoma or renal cell carcinoma using high-dose bolus interleukin-2. JAMA 271(12): 907–913 8120958

[bib15] Sleijfer DT, Janssen RA, Buter J, de Vries EG, Willemse PH, Mulder NH (1992) Phase II study of subcutaneous interleukin-2 in unselected patients with advanced renal cell cancer on an outpatient basis. J Clin Oncol 10: 1119–1123 1607917 10.1200/JCO.1992.10.7.1119

